# Health beliefs and behaviors of livestock industry workers regarding Crimean-Congo hemorrhagic fever in Northwest of Iran

**DOI:** 10.1186/s12913-022-07487-4

**Published:** 2022-01-18

**Authors:** Malek Abazari, Davoud Adham, Abedin Saghafipour, Zahra Taheri-Kharameh, Abbas Abbasi-Ghahramanloo, Javad Asadollahi, Amin Babaei Pouya, Eslam Moradi Asl

**Affiliations:** 1 Department of Public Health, School of Health, Ardabil University of Medical Sciences, Ardabil, Iran; 2 Department of Public Health, Faculty of Health, Qom University of Medical Sciences, Qom, Iran; 3 Department of Operating Room, School of Allied Medical Sciences, Qom University of Medical Sciences, Qom, Iran; 4 Department of Occupational Health, School of Health, Ardabil University of Medical Sciences, Ardabil, Iran

**Keywords:** Disease-preventive behaviors, Health belief model, Crimean-Congo hemorrhagic fever, Butchers, Ardabil

## Abstract

**Background:**

Crimean-Congo hemorrhagic fever (CCHF) is an acute, feverous disease that is caused by tick bites or humans’ direct contact with the blood and tissues of infected livestock and humans. The transmission of the disease is also possible via human-to-human contacts and nosocomial transmission is well described. The majority of patients suffering from this disease are slaughterhouse workers (including butchers), farmers, veterinarians and hospital staff. Thus, this study aimed to investigate the health behaviors of butchers regarding CCHF and study factors affecting such behaviors based on the health belief model.

**Methods:**

This is a descriptive cross-sectional study conducted on 500 butchers in Ardabil Province in 2020 by a multistage sampling method. The participants of the study completed the researcher-made questionnaire of health belief model and health behaviors model relevant to CCHF. The collected data were then analyzed by descriptive statistical tests and linear regression analysis.

**Results:**

The mean (SD) age of the participants was 44.4 (10.5) years, and 96% were males. Only 11.1% of the participants displayed acceptable disease-preventive behaviors. The validity and reliability of the developed questionnaire were confirmed. The results of the exploratory factor analysis showed that the constructs of the model explained 84% of the total variance. The results of the study revealed that among the variables of the health belief model, perceived susceptibility (*p*-value = 0.006, β = 0.152) and perceived barriers (*p*-value = 0.023, β = 0.14) were the strongest factors predicting disease-preventive behaviors regarding CCHF.

**Conclusion:**

The results of the study showed that the health belief model can predict preventive behaviors for CCHF. Therefore, designing and executing interventions based on the results of this study may encourage such preventive behaviors in butchers.

## Background

Crimean-Congo hemorrhagic fever (CCHF) is an acute, feverous disease [1] caused by a tick-borne virus of the Nairoviridaefamily [2]. It is an arboviral disease that is transmissible from arthropods [3] and is transferred from hard-bodied ticks from the genus Hyalomma to humans [4, 5]. CCHF was first diagnosed in the Crimean Peninsula, Ukraine, in 1944; a decade later, a similar disease with the same symptoms was reported in the Republic of the Congo in 1956, hence the name of the disease as CCHF [6–8].

One of the most frequent types of transmission for the disease is tick bites. It can also be transmitted to humans by coming into contact with the blood/tissues of infected wild animals and livestock as well as infected persons. The majority of patients suffering from this disease are butchers, slaughterhouse workers, farmers, veterinarians and hospital staff [9]. Sheep, cow and other domestic animals are the reservoirs of Hyalomma ticks, which if infected, they show no clear symptoms, hence the difficulty of the diagnosis of the disease in animals.

Previous studies emphasized that occupation related high risk behaviors can increase the risk of CCHF more than the personal awareness and performance of the individuals (10, 11). Among workers in livestock industry and slaughterhouse butchers typical behaviors include eating raw liver, holding the knife in the mouth while dressing animal and not wearing appropriate work clothes and boots [9].

The geographical distribution of the disease depends on the distribution of the vector: hard-bodied tick Hyalomma [12]. This virus has been reported from more than 30 African, Eastern European, Middle Eastern and Asian countries [13] including Iran’s neighboring countries such as Afghanistan, Pakistan, Iraq, Turkey, Arabian countries as well as Kazakhstan and Uzbekistan [14]. According to a report by the World Health Organization in 2008 and the geographical dispersion map of the disease in 2015, Iran is located in the endemic belt for the disease [15, 16].

The highest incidence of the disease has been reported in spring and summer, especially in July due to the activity of the vector ticks, and the lowest incidence has been reported in autumn [17]. Based on standard health protocols, repulsive substances and safe acaricides are used [18, 19]. Observing health regulations and exhibiting healthy behaviors result from proper education and awareness and can prevent CCHF [18]. The health belief model can help understand preventive behaviors better and be employed as an effective model in educational pogroms for occupational injuries [20]. Based on this model, people display appropriate behaviors and reactions to health regulations and preventive measures only when they feel they are exposed to a real danger (perceived susceptibility) and this danger is seriously threatening them (perceived severity); thus, they start to believe that changing their behavior is beneficial (perceived benefits) and that they are able to remove barriers in their way of exhibiting correct health behaviors (perceived barriers). Self-efficacy means how a person judges and evaluates their own abilities to perform a task. Although a few studies have been carried out in Iran on health workers’ awareness and performance regarding CCHF [21, 22], no study has specifically focused on butchers’ health beliefs and preventive behaviors in terms of CCHF. Hence, given the importance of their behavior in preventing Crimean-Congo infection, this study aimed to investigate butchers’ preventive behaviors regarding CCHF in Ardabil Province.

## Method

### Study design and setting

**Fig. 1:**
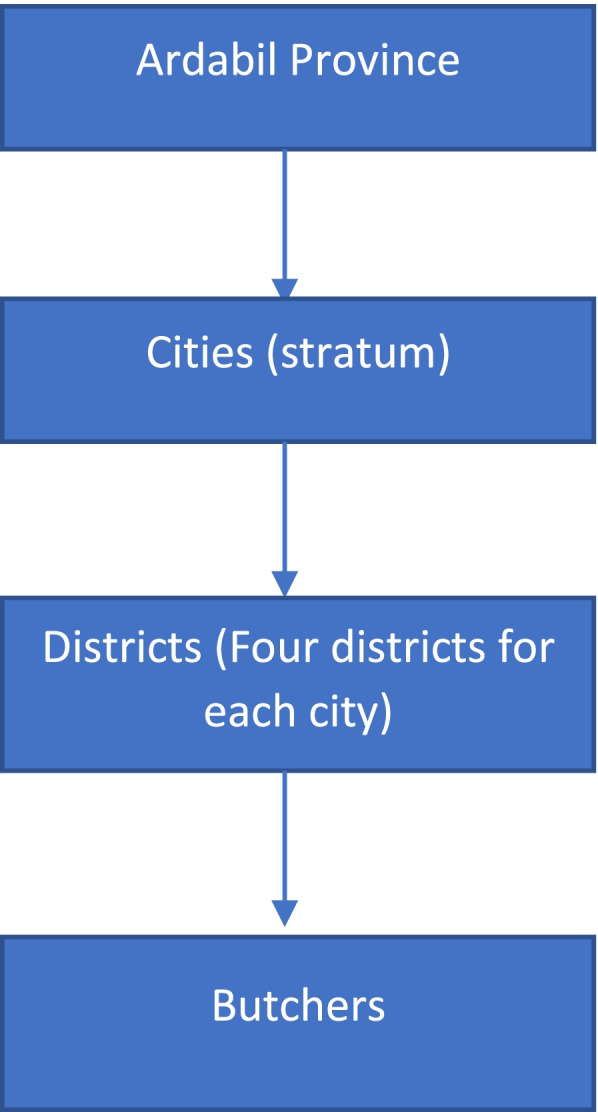
Multistage sampling scheme of the study

This is a descriptive-analytical cross-sectional study including all meat distribution centers in Ardabil Province, Iran, conducted based on a multistage sampling method. In the initial stage, each city in the province was regarded as a stratum; then, in the second stage, each city was divided into four classes, and the required sample was collected from each class based on the convenience sampling method (Fig. 1). A total of 500 butchers working in the livestock and meat distribution industry in Ardabil Province were interviewed concerning CCHF.

### Instrument

The instrument of the study was a standard questionnaire consisting of two sections. The first section comprised participants’ demographic information, and the second section was the health belief scale including 30 questions in six parts: perceived susceptibility construct (four items), perceived severity construct (five items), perceived benefits construct (five items), perceived barriers (five items), perceived self-efficacy (four items) and behavior construct (seven items).

To prepare the scale, a thorough literature review was first conducted according to the main keywords of the study. Then, by designing the health belief model structures, a questionnaire was developed. The reliability of the scale was assessed by Cronbach-alpha, and the validity of the scale was measured in terms of content and construct validities. Ten health education and entomologist experts were asked to assess the questionnaire based on grammatical criteria, necessity, importance, and the placement of phrases in their proper place and were required to provide feedback. The expletory factor analysis (EFA) was implemented to test the construct validity.

### Statistical analysis

Statistical analyses were performed with SPSS IBM-20 software. The significance level was set at *p* ≤ 0.05. For descriptive statistics, mean ± standard deviation (SD) and percentages were used. The Kolmogorov–Smirnov test was used to examine the normality of quantitative variables in the samples. One-way analysis of variance was employed for the comparison of continuous variables among groups (more than 2 categories), and students’ tests were used for the comparison of quantitative variables between the two categories. The health behavior patterns of CCHF patients were derived using Principal Component Analysis (PCA) as a type of factor analysis procedure. Kaiser–Meyer–Olkin (KMOtest) and Bartlett's test of sphericity were used to assess the suitability of running PCA. The sampling adequacy and inter-correlation of factors were supported by KMO value > 0.8 and Bartlett's test of sphericity < 0.001, respectively. Factors were retained based on an eigenvalue of > 1 for the screen plot. Then, Varimax rotation was applied to review the correlations among variables and factors.

## Results

The mean (SD) age of the participants of the study was 44.4 years (10.5). The participants’ age ranged from 17 to 74 years old. Around 52% of the participants lived in cities, and 96% were males; 86.2% were above level 1 in terms of education, and 82.9% were married. The mean (SD) work experience among them was 14.5 years (9.9). The lowest work experience was one year and the highest was 57 years.

The result of the validity of the questionnaire was assessed in terms of content validity. The content validities were as follows: 100% for 15 items, 85% for five items and 80% for eight items. The total validity of the questionnaire was 91.6%. The reliability of the questionnaire was measured based on the Cronbach-alpha index. The total reliability of the questionnaire was 92%: 98.6 for perceived susceptibility construct, 91.6% for perceived severity construct, 77.4% for perceived benefits construct, 88.2% for perceived barriers construct, 75% for perceived self-efficacy construct and 90.5% for behavior construct.

Table 1 demonstrates the results of factor analysis showing the number of factors. The results of the KMO test equaled 0.889 proving that the number of samples for running factor analysis was adequate (KMO ˃ 0.8). The result of Bartlett’s test of sphericity was also significant (p ˂ 0.05) showing that factor analysis was appropriate for detecting the construct and factorial model and that the coefficient matrix of variables in the population formed a unified matrix. In this table, only factors with the special value of 1 or higher were included. As observed in Table 2, six factors were extracted from the questionnaire. The cumulative percentage equaled 83.7% meaning that six factors explained 84% of the total variance.

**Table 1:** Factor loading matrix of groups for CCHF health behaviors in Ardabil Province

Group	1	2	3	4	5	6
Eigenvalue	4.38	3.05	1.83	1.45	1.07	1.03
Because I am cautious, I am not infected with CCHF	**.550**					
CCHF mostly affects inexperienced people; I am experienced, so I am not infected	**.367**					
I do not wear glasses while slaughtering livestock, so I may be infected with CCHF	**.488**					
I do not wear a mask while slaughtering livestock, so I may be infected with CCHF	**.498**					
In my opinion, CCHF is a fatal disease		**.581**				
In my opinion, CCHF can only cause a slight fever, so it is not very serious		**.542**				
In my opinion, CCHF causes long-term hospitalization		**.312**				
In my opinion, a person suffering from CCHF can be recovered		**.628**				
In my opinion, CCHF treatment costs a lot of money		**.446**				
If I wear a mask while slaughtering livestock, I will not contract CCHF			**.612**			
If I wear suitable glasses while slaughtering livestock, I will not contract CCHF			**.610**			
If I wear gloves while slaughtering livestock, I will not contract CCHF			**.557**			
If I am cautious while slaughtering or coming into contact with livestock, I will not contract CCHF			**.421**			
If I am not bitten by ticks while slaughtering, I will not contract CCHF			**.447**			
It is hard for me to use gloves while slaughtering livestock				**.538**		
I could not wear glasses while slaughtering				**.464**		
It is difficult for me to wear a mask while slaughtering because it causes shortness of breath				**.600**		
While slaughtering livestock, wearing protective work clothes and boots is time consuming				**.452**		
While slaughtering, there is no place to hold my knife, so I have to hold my knife in my teeth				.**400**		
I can easily wear glasses while slaughtering					**.635**	
I can always use gloves to touch and slaughter livestock					**.647**	
I can wear a proper mask while slaughtering					**.541**	
I can easily wear boots and work clothes before slaughtering					.**448**	
Do you have a history of contact with carcasses, blood, or raw red meat?						.**605**
Do you always use gloves while slaughtering livestock or coming into contact with blood and carcasses?						.**500**
Do you always wear a mask while slaughtering livestock or coming into contact with blood and carcasses?						.**557**
Do you always wear appropriate glasses while slaughtering livestock or coming into contact with blood and carcasses?						.**654**
Do you always wear protective work clothes and boots while slaughtering livestock or coming into contact with blood and carcasses?						.**381**
Do you always carry a knife in your teeth while slaughtering livestock or coming into contact with blood and carcasses?						.**375**
Do you have a history of physical contact with ticks?						.**529**
Total variance	29.320%	20.001%	12.220%	9.659%	7.127%	5.077%

^*^Absolute factor loading values < 0.30 for the six patterns were excluded for simplicity

**Table 2:** Relationship between HBM constructs and health behaviors of CCHF with demographic variables in Ardabil Province

Variable	Mean (SD)	Living place		Marital		Education			
		Village	Urban	Single	Married	Illiterate	Under Associate Degree	Associate Degree	Above Associate Degree
Perceived susceptibility	9.4(2.5)	9.3(2.3)	9.4(2.5)	9.6(2.2)	9.3(2.5)	8.36(1.96)	8.89(2.37)	10.09(1.95)	10.92(2.24)
		0.683		0.366		0.001			
Perceived severity	12.1(1.6)	12(1.5)	12.2(1.7)	11.9(1.9)	12.51(1.4)	9.89(2.63)	11.35(1.81)	11.89(1.99)	13.21(2.02)
		0.173		.003		0.001			
Perceived benefits	10.6(2.9)	10.7(2.7)	10.5(2.9)	10.9(2.7)	10.5(2.8)	9.01(2.16)	9.94(2.75)	10.80(2.85)	11.71(3.11)
		0.383		0.339		0.001			
Perceived barriers	14(4.4)	14.2(5.4)	13.8(3.1)	14.3(2.7)	13.9((4.6)	9.50(2.80)	13.07(4.81)	14.05(3.11)	16.50(2.51)
		0.341		0.487		0.001			
Self-efficacy	8.7(3.2)	8.3(2.4)	9.2(3.8)	9.2(2.7)	8.6(3.3)	7.63(2.10)	8.58(2.76)	9.48(2.24)	12.30(2.68)
		0.006		0.160		0.001			
Behavior	11.1(1.2)	9.48(1.2)	11.2(1.2)	11.3(0.9)	11.1(1.3)	8.82(1.88)	9.46(1.19)	11.41(1.41)	13.69(1.60)
		0.008		0.113		0.001			
Total	66(5.9)	65.6(5.6)	66.3(6.2)	67.2(6.1)	65.7(5.9)	52.96(7.14)	61.31(6.63)	67.74(5.14)	77.35(7.19)
		0.210		.049		0.001			

The mean and SD of the constructs of the health belief model and preventive behaviors are depicted in Table 2. There was a significant relationship between living location and self-efficacy and behavior as those butchers living in the city scored higher compared to those living in rural areas. The marital status was only significantly related to perceived severity because married butchers scored higher in this construct. Education level was significantly related to all constructs as an increase in the level of education increased the scores in all constructs. Similarly, the score of preventive behavior had a significant relationship with education level because an increase in education level raised the scores in preventive behaviors.

Table 3 depicts correlations between the constructs of the health belief model and preventive behaviors regarding CCHF. As observed in Table 3, all constructs except perceived benefits had a significant correlation with preventive behaviors.

**Table 3:** The relationship between health behaviors of CCHF and HBM constructs in Ardabil Province

Variable	1	2	3	4	4	5
1.Perceived susceptibility	1					
2.Perceived severity	.024	1				
3.Perceived benefits	-.295**	.090	1			
4.Perceived barriers	-.050	-.014	-.174**	1		
5.Self-efficacy	-.051	.194**	.188**	-.517**	1	
6.Behavior	.122*	.113*	-.006	-.180**	.153**	-.122*

The result of regression analysis of the constructs of HBM in predicting the preventive behaviors, using the Enter method, revealed that of all variables of this model, perceived susceptibility and perceived barriers were the most important and strongest factors related to behaviors that could prevent CCHF (Table 4).

**Table 4:** The results of the multiple linear regression analysis of the HBM constructs regarding health behaviors of CCHF in Ardabil Province

Variable	B	Std. Error	Beta	*P* value
Perceived susceptibility	.082	.030	.152	.006
Perceived severity	.078	.042	.100	.062
Perceived benefits	-.032	.026	-.068	.223
Perceived barriers	-.060	.026	-.140	.023
Self-efficacy	.025	.023	.064	.295
Constant	11.841	.721	----	.000

## Discussion

CCHF is one of the most frequent types of occupational diseases amongst veterinarians, butchers and slaughterhouse workers [7, 20]. This study investigated the preventive behaviors of and factors affecting such behaviors in 500 butchers working in butcheries and slaughterhouses in all rural and urban areas of Ardabil Province. Although few similar studies have been carried out in the west of Iran [22] and Turkey [21], this study was the first on its kind that investigated the health beliefs and preventive behaviors of butchers regarding CCHF in this region.

The findings of this study demonstrated that the preventive behaviors of butchers and livestock workers regarding CCHF were not generally acceptable. Other studies have also reported that awareness and attitudes in this regard are less than 10% [23] and emphasized that such workers need periodical education. In another study in the west of Iran, it was found that the awareness and performance of slaughterhouse workers and veterinarians raised significantly after educational courses [12].

Moreover, corresponding with the findings of previous research, it was found that preventive behaviors had significant correlations with perceived susceptibility construct and perceived barriers construct. For instance, Barati et al. found a significant, positive correlation between behavioral intention and perceived threat [24]. Also, Jiang observed that perceived threat regarding SARS preventive behaviors was the strongest predictor of behavior [25].

In this study we identified six dimensions from the questionnaire. These were perceived susceptibility, perceived severity, perceived benefits, perceived barriers, self-efficacy and behavior. The cumulative percentage equaled 83.7% meaning that six factors explained 84% of the total variance. Generally, people have good reactions to health messages and preventive programs when they feel that they are at serious risk (perceived susceptibility); it is just then that they perceive the benefits of changing their behavior (perceived benefit) and remove easily the barriers to these changes (new and healthy preventive behaviors) and become confident whether to do or not to do a behavior (self-efficacy). It is in this situation that educational interventions and programs are likely to be effective [26, 27]. By considering different dimension of health beliefs of butchers, decision and policy makers could design and implement different educational programs toward prevention of CCHF in Ardabil.

According to the results of this study, the living location was the only variable among demographic ones that had a significant relationship with perceived self-efficacy and preventive behaviors. The marital status of the participants was only significantly correlated with perceived severity. The most important factor related to health behavior constructs and preventive behaviors was the educational level of the butchers that had significant correlations with all constructs (*p* ˂ 0.05). In line with the findings of the current study, previous literature has revealed that education and literacy significantly affect awareness, performance, attitude and behavior [28, 29].

Nevertheless, this study has some potential limitations. First, this study is a cross-sectional study and thus it cannot demonstrate the causal relationships among the variables of the study. Moreover, only a self-reported questionnaire was used to evaluate the behaviors of butchers regarding CCHF because it was not possible to observe the CCHF behaviors of butchers objectively.

## Conclusion

According to the results of the study, perceived susceptibility and perceived barriers were the strongest factors predicting the exhibition of preventive behaviors regarding CCHF. Thus, designing and executing appropriate interventions based on the findings of this study can encourage such behaviors in slaughterhouse butchers and workers.

## Data Availability

The data that support the findings of this study are available on request from the corresponding author.

## Abbreviations

CCHF: Crimean-Congo hemorrhagic fever
SD: Standard deviation
PCA: Principal Component Analysis
